# Clinical Images in Emergency Medicine: Man With Foot Pain and Indifference

**DOI:** 10.1016/j.acepjo.2025.100164

**Published:** 2025-05-08

**Authors:** Alex Y. Koo, Susan R. O’Mara

**Affiliations:** 1 Department of Emergency Medicine, Washington Hospital Center, Washington, DC, USA; 2 Department of Clinical Emergency Medicine, Georgetown University School of Medicine, Washington, DC, USA

**Keywords:** necrotizing soft tissue infections, hemorrhagic bullae, osteomyelitis, la belle indifference

## Patient Presentation

**Figure 1:**
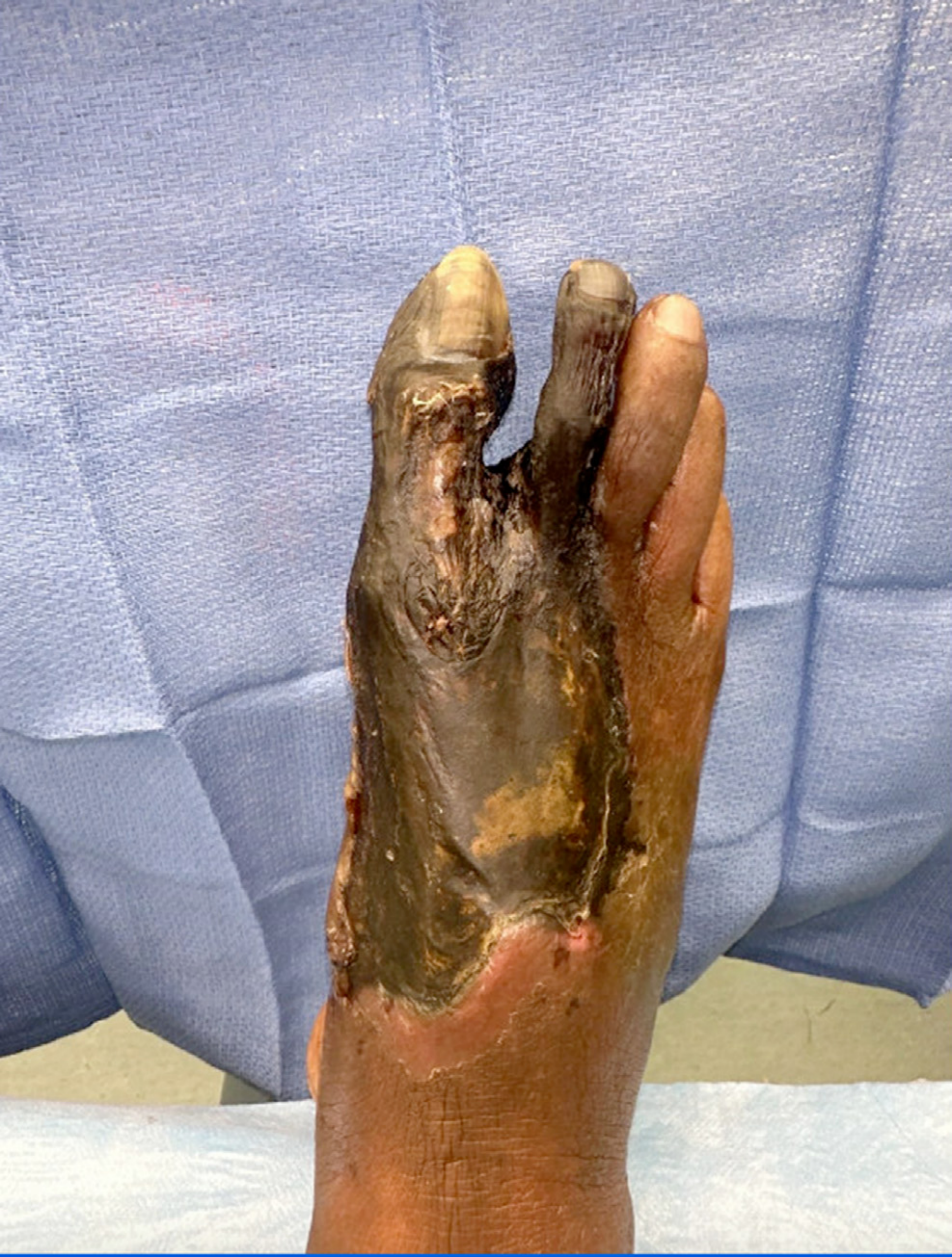
Right foot with wet and dry gangrene.

**Figure 2:**
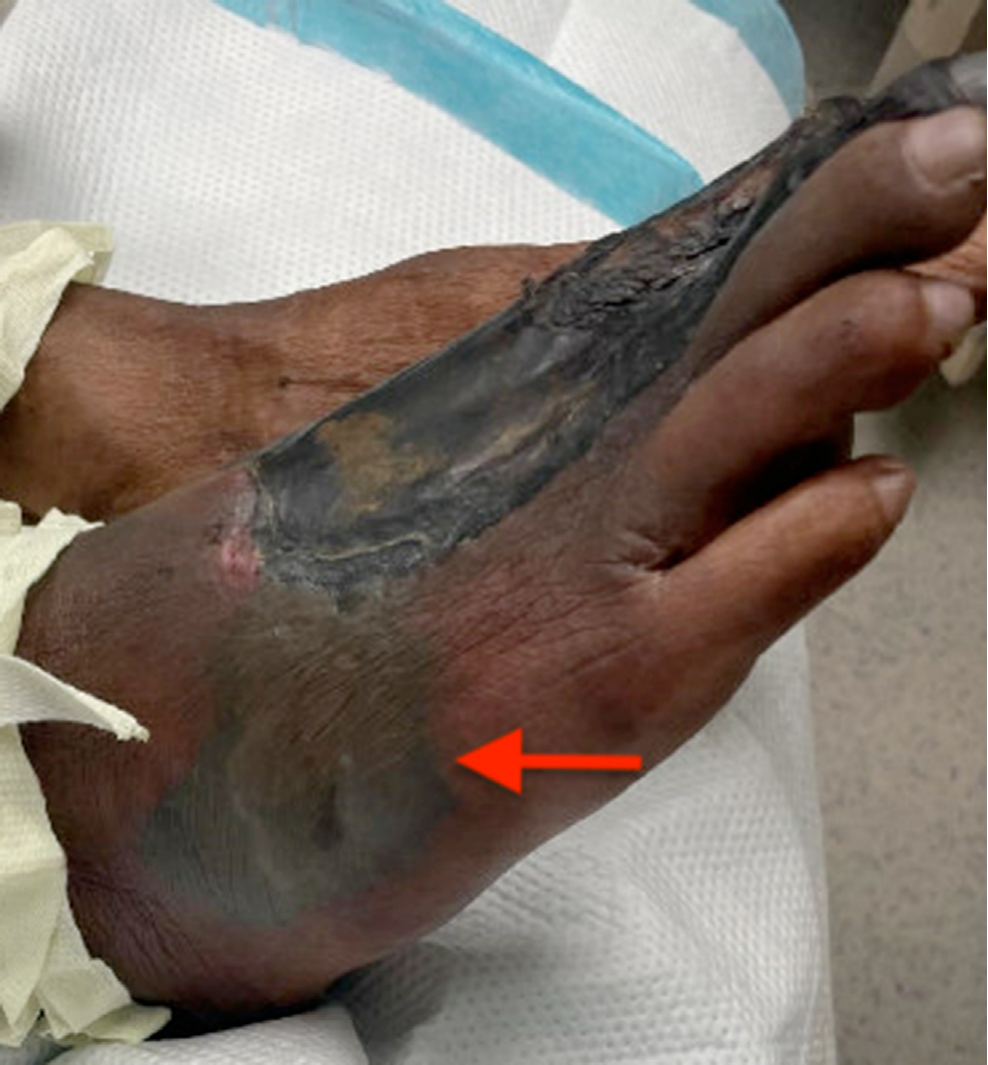
Right foot with hemorrhagic bullae (red arrow).

An 83-year-old male with a history of hypertension and tobacco use presented with right foot pain of unclear duration, reporting its presence for “maybe a couple weeks – or two months.” He was alert and oriented with normal vital signs. Examination of the foot revealed areas of indurated tissue necrosis and hemorrhagic bullae that were minimally painful to touch (Figs 1 and 2). The patient seemingly was indifferent to the concerning appearance of his extremity asking, “Can I just get something for the pain and get out of here?”

## Diagnosis: Necrotizing Soft Tissue Infection With Osteomyelitis of the Foot

**Figure 3:**
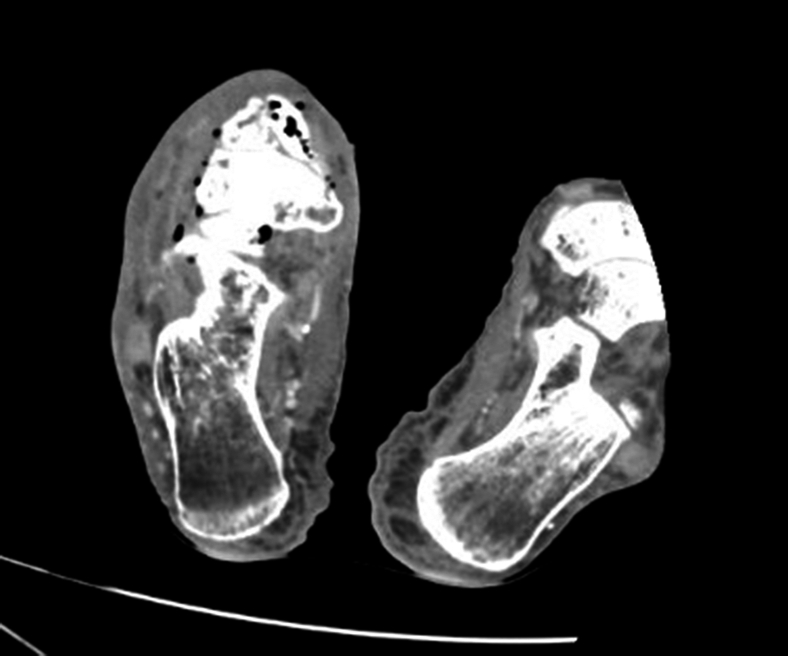
Axial computed tomography right lower extremity with air and stranding in deep tissues.

**Figure 4:**
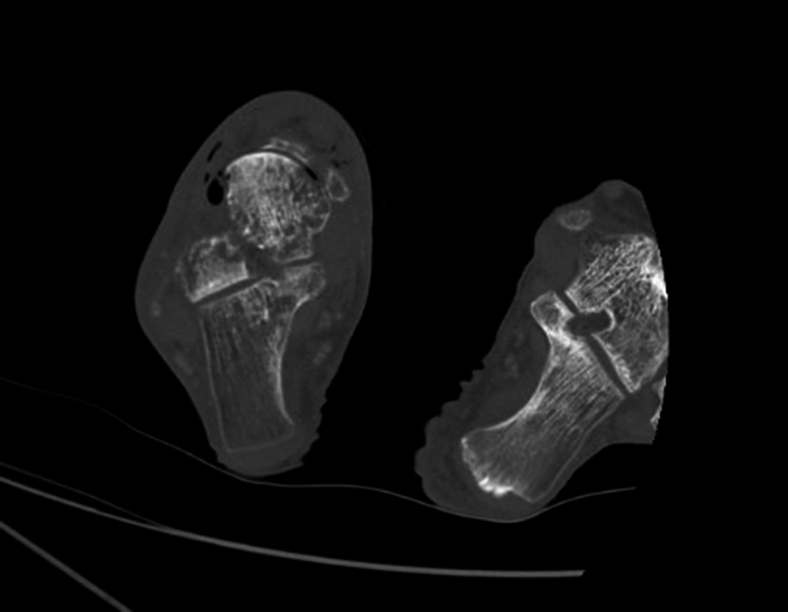
Axial computed tomography right lower extremity with cortical destruction of talar and midfoot bones.

Computed tomography revealed soft tissue gas of the hind- and midfoot and multifocal osteomyelitis involving the talar head and midfoot (Figs 3 and 4). He underwent right ankle disarticulation with deep tissue drainage culture that grew *Morganella morganii*.

Early necrotizing soft tissue infections present with erythema, pain, and swelling, making it difficult to distinguish from cellulitis. As infection progresses, the blood supply of overlying skin is stripped, and erythema progresses to hemorrhagic bullae with extreme local tenderness.^1^ Hemorrhagic bullae have been cited to be >90% specific for a necrotizing soft tissue infection.^2^ Later stages will have appreciable crepitus and systemic signs of sepsis.

“La belle indifference,” defined as a paradoxical absence of distress despite a serious medical illness, has been described in a subset of patients with necrotizing infections.^3^ Although not well studied, it is theorized that anesthesia of the skin due to necrosis may attribute to this phenomenon.^4^

## Funding and Support

By *JACEP Open* policy, all authors are required to disclose any and all commercial, financial, and other relationships in any way related to the subject of this article as per ICMJE conflict of interest guidelines (see www.icmje.org). The authors have stated that no such relationships exist.

## Conflicts of Interest

All authors have affirmed they have no conflicts of interest to declare.

## References

[1] Kiat H.J., En Natalie Y.H., Fatimah L. (2017). Necrotizing fasciitis: how reliable are the cutaneous signs?. J Emerg Trauma Shock.

[2] Fernando S.M., Tran A., Cheng W., Rochwerg B. (2019). Necrotizing soft tissue infection: diagnostic accuracy of physical examination, imaging, and LRINEC score: a systematic review and meta-analysis. Ann Surg.

[3] Stone J., Smyth R., Carson A., Warlow C., Sharpe M. (2006). La belle indifférence in conversion symptoms and hysteria: systematic review. Br J Psychiatry.

[4] Raam R., Moran G.J., Jhun P., Herbert M. (2016). Worms and flesh-eating bacteria? The worst day of your life. Ann Emerg Med.

